# Aqueous Extract of Black Maca Prevents Metabolism Disorder via Regulating the Glycolysis/Gluconeogenesis-TCA Cycle and PPARα Signaling Activation in Golden Hamsters Fed a High-Fat, High-Fructose Diet

**DOI:** 10.3389/fphar.2018.00333

**Published:** 2018-04-06

**Authors:** Wenting Wan, Hongxiang Li, Jiamei Xiang, Fan Yi, Lijia Xu, Baoping Jiang, Peigen Xiao

**Affiliations:** ^1^Institute of Medicinal Plant Development, Chinese Academy of Medical Sciences and Peking Union Medical College, Beijing, China; ^2^Key Laboratory of Bioactive Substances and Resources Utilization of Chinese Herbal Medicine, Ministry of Education, Beijing, China; ^3^School of Sciences/Key Laboratory of Cosmetic, China National Light Industry, Beijing Technology and Business University, Beijing, China

**Keywords:** maca (*Lepidium meyenii* Walpers), metabolism disorder, metabolomics, glycolysis/gluconeogenesis-TCA cycle, lipid metabolism

## Abstract

Maca (*Lepidium meyenii* Walpers) has been used as a dietary supplement and ethnomedicine for centuries. Recently, maca has become a high profile functional food worldwide because of its multiple biological activities. This study is the first explorative research to investigate the prevention and amelioration capacity of the aqueous extract of black maca (AEM) on high-fat, high-fructose diet (HFD)-induced metabolism disorder in golden hamsters and to identify the potential mechanisms involved in these effects. For 20 weeks, 6-week-old male golden hamsters were fed the following respective diets: (1) a standard diet, (2) HFD, (3) HFD supplemented with metformin, or (4) HFD supplemented with three doses of AEM (300, 600, or 1,200 mg/kg). After 20 weeks, the golden hamsters that received daily AEM supplementation presented with the beneficial effects of improved hyperlipidemia, hyperinsulinemia, insulin resistance, and hepatic steatosis *in vivo*. Based on the hepatic metabolomic analysis results, alterations in metabolites associated with pathological changes were examined. A total of 194 identified metabolites were mapped to 46 relative metabolic pathways, including those of energy metabolism. In addition, via *in silico* profiling for secondary maca metabolites by a joint pharmacophore- and structure-based approach, a compound-target-disease network was established. The results revealed that 32 bioactive compounds in maca targeted 16 proteins involved in metabolism disorder. Considering the combined metabolomics and virtual screening results, we employed quantitative real-time PCR assays to verify the gene expression of key enzymes in the relevant pathways. AEM promoted glycolysis and inhibited gluconeogenesis via regulating the expression of key genes such as *Gck* and *Pfkm*. Moreover, AEM upregulated tricarboxylic acid (TCA) cycle flux by changing the concentrations of intermediates and increasing the mRNA levels of *Aco2*, *Fh*, and *Mdh2*. In addition, the lipid-lowering effects of AEM in boththe serum and liver may be partly related to PPARα signaling activation, including enhanced fatty acid β-oxidation and lipogenesis pathway inhibition. Together, our data demonstrated that AEM intervention significantly improved lipid and glucose metabolism disorder by regulating the glycolysis/gluconeogenesis-TCA cycle and by modulating gene expression levels involved in the PPARα signaling pathway.

## Introduction

Metabolism disorder are major symptoms in several pathological phenomena, like atherosclerotic cardiovascular disease and type 2 diabetes. Excess energy intake and concomitant obesity are the major drivers of this syndrome. By alleviating metabolism disorder, the onset of diabetes, atherosclerotic cardiovascular disease, and other associated comorbidities might be postponed. Currently, the recommended treatments for metabolic impairments are lifestyle changes, such as medicinal plant supplementation, caloric restriction, healthier foods, and increased physical activity (Grundy, 2016). Clinical trials have shown that medicinal plant supplements can reduce the likelihood of early-stage metabolism disorder progressing to type 2 diabetes (Jia et al., 2015). Nutraceutical interventions aimed at correcting metabolism disorder may be a promising option, but thus far, few natural products have been investigated. Traditional and folk edible herbal treatments are gaining attention for their health benefits and low toxicity and might be suitable for long-term supplementation in metabolism disorder.

Maca (*Lepidium meyenii* Walpers), known as “Peruvian ginseng,” is an annual herbaceous plant of the Brassicaceae family, growing at elevations of over 4,000 m in the Andes region. This herb grows in a habitat with a harsh climate, such as intense cold, extremely intense sunlight, and strong winds (Gonzales et al., 2006). Maca is traditionally consumed fresh or dehydrated after being boiled in water or milk and can be made into juices, cocktails, alcoholic beverages, or maca coffee (Campos et al., 2013). Since the time of the Incas, maca has been used as an energizing and revitalizing food additive; has been recommended for malnutrition, convalescence, memory loss, fatigue, mental weakness, and insomnia; and has been used to regulate menstruation and lessen menopausal symptoms (Henry et al., 2016). Recently, maca attracted global attention when it was listed as safe to eat by the Food and Agriculture Organization. After several years of research and development, the safety and various pharmacological effects of maca have been increasingly recognized.

Metabolomic approaches have been widely applied to study metabolic changes in response to disease or interventions by the analysis of various biological samples, including blood, urine, and tissue. The power of metabolomic approaches has already been used to create profiles that create biomarkers for obesity-associated insulin resistance and to identify metabolites that correlate with diabetes under subclinical conditions, which is key for early detection and management (Sallese and Zhu, 2017). Recent advances in metabolomics suggest that it is possible to not only use this analytical technique to assess the holistic effects of nutritional intervention in metabolism disorder but also investigate the compound–target interactions and to analyze involved metabolic pathways (Puiggròs et al., 2015; Le et al., 2016; Li et al., 2016). Moreover, the natural product is delicate and complex, and its pharmacodynamics characteristics are regulated by different functional groups (Ma et al., 2017). As is known, the functional food discovery process based on natural products is risk-, time-, and cost-intensive. Several studies have confirmed the high success rates of computer-assisted tools, such as virtual screening, to increase the efficiency and efficacy of discovering lead structures in medicinal chemistry (Kitchen et al., 2004; Harvey, 2008). The combination of metabolomic approaches and virtual screening with multivariate data analysis can be used to evaluate pharmacodynamic effects and to understand the molecular mechanisms of the signaling pathways.

This research is the first to study the capacity of an aqueous extract of black maca (AEM) to prevent and improve high-fat, high-fructose diet (HFD)-induced metabolism disorder in golden hamsters. To assess the potential health benefits of AEM, a 20-week intervention study was conducted. We took advantage of recent developments in pharmacophore- and structure-based screening approaches to predict the potential active protein targets of AEM. In addition, mass spectrometry was used to obtain the metabolomic profile of the liver following AEM treatment, and several changes in the metabolic pathway were identified. Building on the success of the *in silico* target prediction and the capacity to quantitatively measure metabolome abundance, we used quantitative real-time PCR (qPCR) to verify the potential underlying molecular mechanisms. These results suggested that AEM may improve metabolism disorder via upregulating the glycolysis/gluconeogenesis-tricarboxylic acid (TCA) cycle pathway and enhancing the expression of genes involved in lipid metabolism.

## Materials and Methods

### Plant Extract

Black maca powder was obtained from TTD International Pty Ltd. (QLD, Australia). An aqueous extract of maca powder was prepared according to traditional methods. The powder (500 g) was placed into a container with 2,000 ml of water, sonicated for 30 min, and then boiled for 2 h. Afterward, the aqueous extract was cooled to room temperature and filtered. Water (500 ml) was added to the filter residue, and the solution was boiled again for 2 h. The two filtrates were combined and concentrated to 416 ml. The concentrate, containing 1,200 mg/ml maca, was placed in small vials and stored in a refrigerator at 4°C until further use. Before the experiments, the concentrate was dissolved in aqueous solution to concentrations of 600 and 300 mg/ml. The nutritional composition of the AEM is shown in **Table 1**.

**Table 1:** Nutritional composition in aqueous extract of black maca (AEM 1,200 mg/kg).

Item	Content
Energy (kJ/100 g)	56.144
Protein (g/100 g)	0.44
Fat (g/100 g)	0.4
Carbohydrate (g/100 g)	1.992
Potassium (mg/100 g)	283.36
Calcium (mg/100 g)	2.6
Ash (g/100 g)	0.368
Water (g/100 g)	396.8


### Animals and Diets

Forty-eight 6-week-old male golden hamsters (Vital River Laboratory Animal Technology Co., Ltd., Beijing, China) were housed in a temperature-controlled (22 ± 2°C) room in our animal center on a 12 h:12 h light–dark cycle with food and water available. The standard chow was composed of 28% protein, 12% lard, and 60% vegetable starch. The HFD contained 30% fructose, 0.5% cholesterol (CHO), 22% lard, 14.5% protein, and 33% vegetable starch. All the animal care procedures and interventions were performed in accordance with the Guidelines and Policies for Animal Surgery under the control of the Chinese Academy of Medical Sciences and Peking Union Medical College, Beijing, China (approval No: SLXD-2016052117) and were approved by the Institutional Animal Use and Care Committee (IACUC). All the animal experiments were conducted based on the recommendations for the care and use of laboratory animals proposed by the National Institutes of Health regulations.

### Experimental Scheme

Golden hamsters were randomly divided into the following six treatment groups, with eight animals in each group:

(1) Control group (standard chow)
(2) HFD group (HFD)
(3) Metformin (Metf) group (daily instillation of 125 mg/kg metformin + HFD)
(4) Low dose of AEM (daily instillation of 300 mg/kg AEM + HFD)
(5) Middle dose of AEM (daily instillation of 600 mg/kg AEM + HFD)
(6) High dose of AEM (daily instillation of 1,200 mg/kg AEM + HFD)

The daily food intake of the golden hamsters in each group was recorded during the 20-week treatment period. The body weights of the golden hamsters in each group were noted weekly. At the end of the 20 weeks, after 12 h of fasting, the hamsters were euthanatized via pentobarbital. Blood samples were then taken from the abdominal aorta, and the serum was separated by centrifugation at 5,000 rpm for 10 min and stored at -20°C. Liver samples and epididymal white adipose tissue (WAT) were dissected at the time of death, sections from the same sample were prepared for histological examination, and liver subsections were frozen in liquid nitrogen for metabolomic analysis. All the serum and hepatic biochemical parameters were determined using appropriate kits (Biosino Bio-technology and Science Incorporation, Beijing, China) according to the manufacturer’s instructions. The serum insulin levels were measured using a radio immunoassay kit (Beijing North Institute of Biological Technology, Beijing, China).

### Determination of Metabolic Parameters and Insulin Sensitivity

The homeostasis model of insulin resistance (HOMA-IR) was used with the following formulas: HOMA-IR = [Fasting insulin level (mU/ml)]^∗^[Fasting serum glucose (mmol/l)]/22.5; and Insulin sensitivity index = 1/[Fasting insulin level (mU/ml)]^∗^[Fasting serum glucose (mmol/l)].

### Histological Examination

Livers and epididymal WAT were removed immediately and immersed in formalin solution for at least 24 h. Then, the samples were embedded in paraffin, and 5-μm-thick slices were sectioned and stained with hematoxylin and eosin (H&E). The images were observed with a light microscope (Olympus IX51, Tokyo, Japan).

### Metabolite Profiling

Liver tissue extraction, pulse-acquire sequence, metabolite identification, and data processing were performed as described previously (Wan et al., 2017). The metabolomic analysis was performed on a Q Exactive Orbitrap (Thermo, United States) equipped with an amide column (Waters, CA, United States). The processed results were imported to MetaboAnalyst 3.0^1^ for analysis.

### *In Silico* Predictions

Through literature reviews, we collected the components and structures of maca (**Supplementary File S1**). All chemistries were used for target fishing with two databases: the PharmaDB database and a similarity ensemble approach (SEAware 1.7; SeaChange Pharmaceuticals, Inc., San Francisco, CA, United States). When PharmaDB was used, the chemical structures were prepared in SD format and converted from a 2D cdx file format to 3D models using Open Babel GUI version 2.3.2 (OpenBableGUI; Chris Morley, United States). Molecular energy was minimized using the Energy Minimization module of Discovery Studio version 4.5 (DS 4.5; Accelrys Inc., San Diego, CA, United States) under the Chemistry at Harvard Macromolecular Mechanics (CHARMM) force field (Yi et al., 2016). All pharmacophore models with the shape of the binding pocket were selected for virtual screening using the default settings of the Ligand Profiler module of DS 4.5. Ligand and pharmacophore fitness characteristics were assessed by the values and shape similarity of the molecules. When using SEAware, molecules were prepared in the SMILES format (Simplified Molecular Input Line Entry System^2^). The 2D structural similarity of each compound to each target’s ligand set was quantified as an expectation value (Keiser et al., 2009). A compound-target-disease table was established via analyzing the hit targets, associated protein and diseases, and the interactions between these parameters.

### RNA Isolation, cDNA Generation, and Quantitative Real-Time PCR (qPCR)

Total RNA was extracted from flash-frozen liver tissue using an RNAiso Plus kit (Takara BIO Inc., Dalian, China) and was reverse-transcribed with a PrimeScript RT reagent kit (Takara BIO Inc., Dalian, China) according to the manufacturer’s instructions. qPCR was performed using SYBR Premix DimerEraser (Takara BIO Inc., Dalian, China) with a Fast Real-time PCR System 7500 (Applied Biosystems 7500 Fast Real-Time PCR System). Reaction conditions were as follows: heating to 95°C in 30 s, followed by 40 cycles at 95°C for 3 s, 55°C for 30 s and 72°C for 30 s. Each cDNA sample was tested with at least three independent replicates to check the data reproducibility. Data were analyzed using the 2^-ΔΔCt^ method. **Supplementary Table S1** contains the list of primers used for qPCR.

### Statistical Analysis

Statistical analyses were performed in GraphPad Prism, Version 5.0 (GraphPad Software Inc., CA, United States) using Student’s *t*-test. The data are expressed as the means ± standard errors of the mean (SEMs). In this paper, significant differences in multiple comparisons were identified at *P* < 0.05, *P* < 0.01, and *P* < 0.001.

## Results

### Effects of AEM on Body Weight, Food Intake, and Food Utilization

After 20 weeks, the golden hamsters in the control group consumed a total of 1,255.05 g of the normal diet; the HFD; Metf; and 300, 600, and 1,200 mg/kg AEM groups consumed 861.17; 922.67; and 969.37, 950.53, and 861.41 g of the HFD, respectively (**Supplementary Figure S1**). **Table 2** shows that food intake was substantially decreased in the HFD-fed golden hamsters compared with that in the hamsters fed a normal diet. The Metf and 300 and 600 mg/kg AEM groups presented with increased food intake, but the food intake of the 1,200 mg/kg AEM group was not affected. The food utilization rate was higher in the HFD group than that in the control group, while the Metf and AEM (300, 600, and 1,200 mg/kg) groups presented a clear, decreasing trend in this parameter (**Table 2**). Moreover, none of the initial body weights were different among the groups. After 20 weeks, compared with the normal diet, the HFD led to greater final body weight gain and a higher fat coefficient in the golden hamsters. The final body weight, total fat mass, and fat coefficient were significantly decreased in the Metf and AEM (1,200 mg/kg) groups compared with the HFD group.

**Table 2:** Effects of aqueous extract of black maca (AEM) and metformin on body weight, food intake, food utilization, and fat and liver coefficients in golden hamsters.

	Control	HFD	Metf	AEM 300	AEM 600	AEM 1200
Initial body weight (g)	102.35 ± 5.75	103.19 ± 5.02	102.77 ± 3.45	103.07 ± 4.99	103.39 ± 5.17	102.08 ± 5.54
Final body weight (g)	215.44 ± 19.55	216.88 ± 13.88	200.22 ± 14.67*	216.80 ± 14.22	215.40 ± 21.07	196.00 ± 19.98*
Body weight gain (g)	112.52 ± 21.11	114.79 ± 14.04	97.74 ± 14.08*	113.73 ± 10.35	112.01 ± 18.97	93.92 ± 20.19*
Food intake (g)	1255.05 ± 1.86	861.17 ± 1.58###	922.67 ± 1.75*	969.37 ± 1.44**	950.53 ± 1.17**	861.41 ± 1.33
Food utilization rate (%)	8.97 ± 1.68	13.33 ± 1.63###	10.59 ± 1.53**	11.73 ± 1.07*	11.78 ± 2.00	10.90 ± 2.34*
Total fat weight (g)	12.05 ± 2.06	13.10 ± 1.76	10.26 ± 1.38**	12.00 ± 1.69	13.02 ± 1.44	10.65 ± 1.40**
Fat coefficient (%)	5.65 ± 0.81	6.06 ± 0.95	5.15 ± 0.63*	5.50 ± 0.59	6.13 ± 0.77	5.59 ± 0.79
Liver weight (g)	6.10 ± 2.26	11.84 ± 1.68###	8.44 ± 0.98***	10.48 ± 0.80	11.38 ± 1.19	9.96 ± 1.35*
Liver coefficient (%)	2.78 ± 0.97	5.45 ± 0.61###	4.30 ± 0.44***	4.94 ± 0.26*	5.44 ± 0.57	5.10 ± 0.52


*Data are presented as the means ± SEMs. ^#^*P* < 0.05, ^##^*P* < 0.01, ^###^*P* < 0.001 compared with the control group; ^∗^*P* < 0.05, ^∗∗^*P* < 0.01, ^∗∗∗^*P* < 0.001 compared with the HFD model group, *n* = 6*

### Effects of AEM Intervention on Liver and Epididymal WAT

**FIGURE 1:**
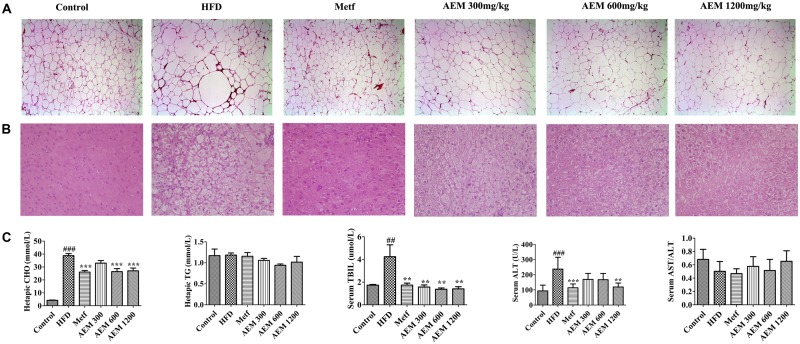
Effects of aqueous extract of black maca (AEM) on the histology of epididymal white adipose tissue and liver and the hepatic lipid profiles in golden hamsters. **(A)** The epididymal white adipose tissue sections were stained with H&E. **(B)** The liver sections were stained with H&E. **(C)** The hepatic cholesterol (CHO), hepatic triglyceride (TG), serum total bilirubin (TIBL), serum aminotransferase (AST), and AST/alanine aminotransferase (ALT) levels were measured. Data are presented as the means ± SEMs. ^#^*P* < 0.05, ^##^*P* < 0.01, ^###^*P* < 0.001 compared with the control group; ^∗^*P* < 0.05, ^∗∗^*P* < 0.01, ^∗∗∗^*P* < 0.0 compared with the HFD model group, *n* = 6.

We evaluated the development of inflammation in epididymal WAT (**Figure 1A**). The histological analysis of the HFD-fed golden hamsters revealed a notable enlargement in adipocyte size (**Supplementary Figure S2**) and increased inflammation. However, AEM (300, 600, and 1,200 mg/kg) and metformin treatment attenuated this trend, resulting in adipocytes with a similar shape and size as those in the control group. In addition, H&E section staining revealed that compared with chow diet-fed golden hamsters, hamsters that received the long-term HFD presented with substantial liver lipid deposition. In contrast, AEM (300, 600, and 1,200 mg/kg) treatment and metformin ameliorated the lipid accumulation in the liver (**Figure 1B**). The liver coefficient is the ratio of the liver mass to the body mass and should be constant under normal conditions. An increased liver coefficient indicates edema, inflammation, or hyperplasia in the liver tissue. **Table 2** shows that the liver weight and liver coefficient were significantly increased in the HFD group compared with the control group, while AEM (300 and 1,200 mg/kg) treatment changed this trend. We further measured the levels of hepatic triglycerides (TGs) and CHO and found that metformin and AEM (600 and 1,200 mg/kg) treatment markedly reduced the liver CHO content and that AEM (300, 600, and 1,200 mg/kg) caused a downward trend in TG levels (**Figure 1C**). In addition, the HFD led to higher serum total bilirubin (TBIL) levels, while the administration of different doses of AEM (300, 600, and 1,200 mg/kg) reversed this trend. Serum aminotransferase (AST) and alanine aminotransferase (ALT) levels reflect the degree of liver damage. The serum ALT levels in the HFD group were increased dramatically, but the values in the different AEM dose groups tended to be close to those of the control group. The AST/ALT ratio also showed that AEM did not damage the liver function of golden hamsters (**Figure 1C**).

### Effects of AEM on Serum Biochemical Parameters

**FIGURE 2:**
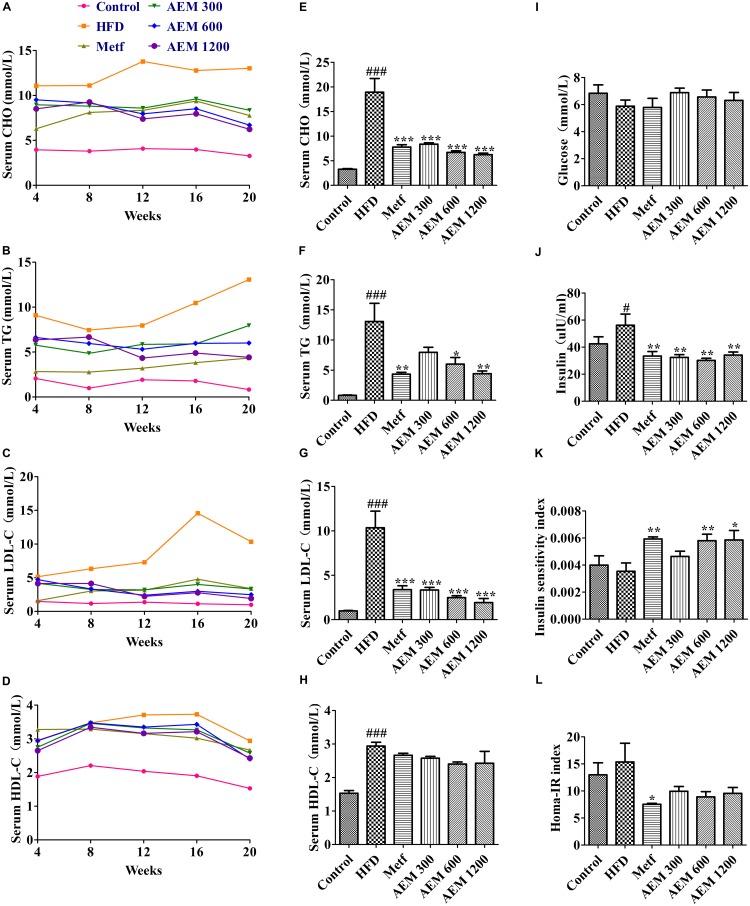
Effects of aqueous extract of black maca (AEM) on the serum lipid, glucose, and insulin profiles in golden hamsters. Over 20 weeks, **(A)** serum cholesterol (CHO), **(B)** serum triglyceride (TG), **(C)** serum low-density lipoprotein CHO (LDL-C), and **(D)** high-density lipoprotein CHO (HDL-C) levels were tested. At the end of 20 weeks, **(E)** serum CHO, **(F)** serum TG, **(G)** serum LDL-C, **(H)** serum HDL-C, **(I)** serum glucose, and **(J)** serum insulin were measured. The calculated insulin sensitivity index **(K)** and HOMA-IR index **(L)** are displayed. Data are presented as the means ± SEMs. ^#^*P* < 0.05, ^##^*P* < 0.01, ^###^*P* < 0.001 compared with the control group; ^∗^*P* < 0.05, ^∗∗^*P* < 0.01, ^∗∗∗^*P* < 0.001 compared with the HFD model group, *n* = 6.

As shown in **Supplementary Figure S3**, compared with the normal diet, the HFD significantly increased the CHO, TG, high-density lipoprotein CHO (HDL-C), and low-density lipoprotein CHO (LDL-C) levels after 4 weeks. These results revealed that the golden hamsters developed significant hyperlipidemia after 4 weeks of the HFD feeding. During the 20 weeks of treatment, the serum TG, CHO, HDL-C, and LDL-C levels were low and stable in the control group but were comparatively much higher and continued to increase in the HFD group. After the AEM supplementation, both treated groups showed clear, dose-dependent decreases in serum lipid levels (**Figures 2A–D**). After 20 weeks, compared with the HFD group, both the AEM-treated groups presented with significant reductions in serum CHO and LDL-C levels (**Figures 2E,G**). Moreover, AEM supplementation (600 and 1,200 mg/kg) significantly reduced TG accumulation; this decreasing trend was also observed in the 300 mg/kg AEM group but was not significant (**Figure 2F**). Serum HDL-C levels were slightly but non-significantly decreased in all the AEM groups (**Figure 2H**).

Glucose and insulin levels were also measured over the course of this study (**Figures 2I–L**). Glucose levels remained unchanged among the HFD and AEM groups (**Figure 2I**). Serum insulin concentrations were significantly lower after 20 weeks of metformin and AEM treatment compared with these levels following the HFD treatment (**Figure 2J**). Compared with the HFD group, among the Metf and AEM (600 and 1,200 mg/kg) groups, the insulin sensitivity index was significantly increased (**Figure 2K**).

### Protein–Ligand Docking

As shown in **Supplementary File S1**, 63 compounds from maca were collected by literature review: 56% of the compounds were alkaloids and macaenes (compounds A1–A35), 33% were glucosinolates (compounds J1–J21), and the others were sterols (compounds S1–S7). We chose six disease categories (obesity, insulin resistance, hyperinsulinemia, hyperglycemia, hyperlipidemia, and diabetes) closely related to glucose and lipid metabolism disorders to validate the medical actions and to identify maca compounds. **Supplementary Table S2** provides an overview of the compound-target-disease relationships. Fructose-1,6-bisphosphatase 1 (F16P1), which acts as a rate-limiting enzyme in gluconeogenesis, was determined by the alkaloids (A1, A12, A14, A16, A17, and A21) and the glucosinolate (J9). The alkaloids (A3, A7, A10, A12, A13, A23, A24, and A27) and the glucosinolates (J4 and J13) identified glycogen synthase kinase-3 beta (GSK3B). The glucosinolate (J10) and alkaloids (A6, A8, A14, A15, and A24) were connected to protein-tyrosine phosphatase 1B (PTN1). Peroxisome proliferator-activated receptor gamma (PPARγ), a key regulator of adipocyte differentiation and glucose homeostasis, was found by the alkaloids (A10, A11, A14, A15, A16, A24, and A25). We found that the glucosinolates (J10, J11, J12, and J14) and (J3, J4, J9, and J14) were associated with sodium/glucose cotransporter 1 (SC5A1) and sodium/glucose cotransporter 2 (SC5A2), respectively. The glucosinolate (J9) and sterols (S1, S2, S4, and S7) were related to acetyl-CoA acetyltransferase (THIL). Glucosinolates (J3, J4, J12, J13, J14, J15, and J16), (J15 and J16), (J4 and J9), and (J11) were separately found with aldose reductase (ALDR), dipeptidyl peptidase 4 (DPP4), corticosteroid 11-beta-dehydrogenase isozyme 1 (DHI1), and liver carboxylesterase 1 (EST1), respectively. The alkaloids (A13), (A7 and A25), and (A13 and A14) were separately involved with the bile acid receptor (FXR), lanosterol synthase (ERG7), and peroxisome proliferator-activated receptor alpha (PPARα), respectively. Oxysterols receptor LXR-beta (LXRβ) interacted with the alkaloid (A24) and sterol (S7); mitogen-activated protein kinase 14 (MK14) was identified by the sterol (S7) and glucosinolates (J4, J9, and J10).

### Metabolomic Analysis and Pathway Impact Analysis

**FIGURE 3:**
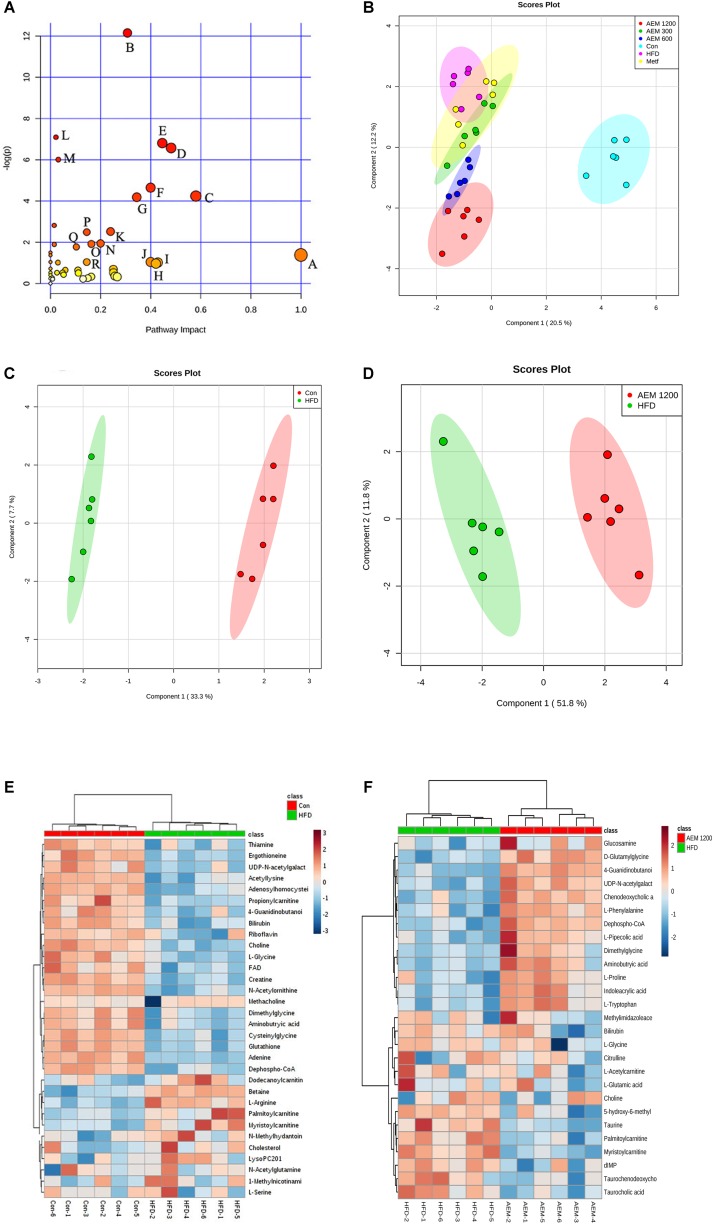
Aqueous extract of black maca (AEM) regulated the disturbed metabolism. **(A)** Summary of pathway analysis with MetPA. A, D-Glutamine and D-glutamate metabolism; B, Arginine and proline metabolism; C, Glyoxylate and dicarboxylate metabolism; D, Glutathione metabolism; E, Nicotinate and nicotinamide metabolism; F, Thiamine metabolism; G, TCA cycle; H, Alanine, aspartate, and glutamate metabolism; I, Taurine and hypotaurine metabolism; J, Ascorbate and aldarate metabolism; K, Cysteine and methionine. **(B)** Orthogonal partial least-squares discriminant analysis (OPLS-DA) score plots for UPLC/MS data of all the groups. OPLS-DA score plots showed differences in the metabolic states in the control group (Con), the HFD model group (HFD), the metformin groups (Metf), the 300 mg/kg AEM group (AEM 300), the 600 mg/kg AEM group (AEM 600), and the 1,200 mg/kg AEM group (AEM 1200). **(C)** OPLS-DA score plot showing the difference in the metabolic state between the control group (Con) and the HFD model group (HFD). **(D)** OPLS-DA score plot showing the difference in the metabolic state between the HFD model group (HFD) and the 1,200 mg/kg AEM group (AEM 1200). **(E)** The heatmap of the correlation analysis of the key metabolites between the control group (Con) and the HFD model group (HFD). **(F)** The heatmap of the correlation analysis of the key metabolites between the HFD model group (HFD) and the 1,200 mg/kg AEM group (AEM 1200), *n* = 6.

By MetaboAnalyst 3.0, a total of 194 identified metabolites were mapped to KEGG metabolic pathways for over-representation and pathway topology analyses. Ultimately, 46 relative metabolic pathways were identified (**Figure 3A**). The pathway impact factor was evaluated by the relative importance of the compounds. Arginine and proline metabolism, glyoxylate and dicarboxylate metabolism, the TCA cycle, and glycolysis/gluconeogenesis had the highest impact factors.

Metabolomics uses multivariate statistical tools on a data set acquired from an MS-coupled method to discriminate systematic variation using a subsequent supervised statistical analysis, such as orthogonal partial least-squares discriminatory analysis (OPLS-DA). This analysis clearly separates a data set into different groups, ultimately screening candidate metabolites for variation (And and Sturm, 2007). To gain an overview of the metabolic contents, OPLS-DA was performed among the different groups. As seen in the OPLS-DA score plot (**Figure 3B**), well-delineated clusters and separation trends among the control, HFD, and AEM-treated groups were observed. The results from the three different AEM dose groups mostly overlapped and significantly deviated from those of the HFD group, indicating that metabolic perturbation occurs and that AEM exerts an intervening effect on this disturbance. In addition, a well-fitted, two component OPLS-DA model was constructed to identify the differential metabolites that respond to the control vs. HFD group and the HFD vs. AEM (1,200 mg/kg) group. **Figure 3C** shows that the metabolic profile of the HFD-fed golden hamsters was fairly different from the profile of the control hamsters. A similar distinction was apparent between the HFD and AEM (1,200 mg/kg) groups (**Figure 3D**). The metabolomic pattern of the key metabolites, those with *P*-values < 0.05, are expressed as squares in the heatmap, which displays the normalized data values using carefully chosen color gradients (**Figures 3E,F**).

### Effects of AEM on Hepatic mRNA Expression Related to Energy and Lipid Metabolism

**FIGURE 4:**
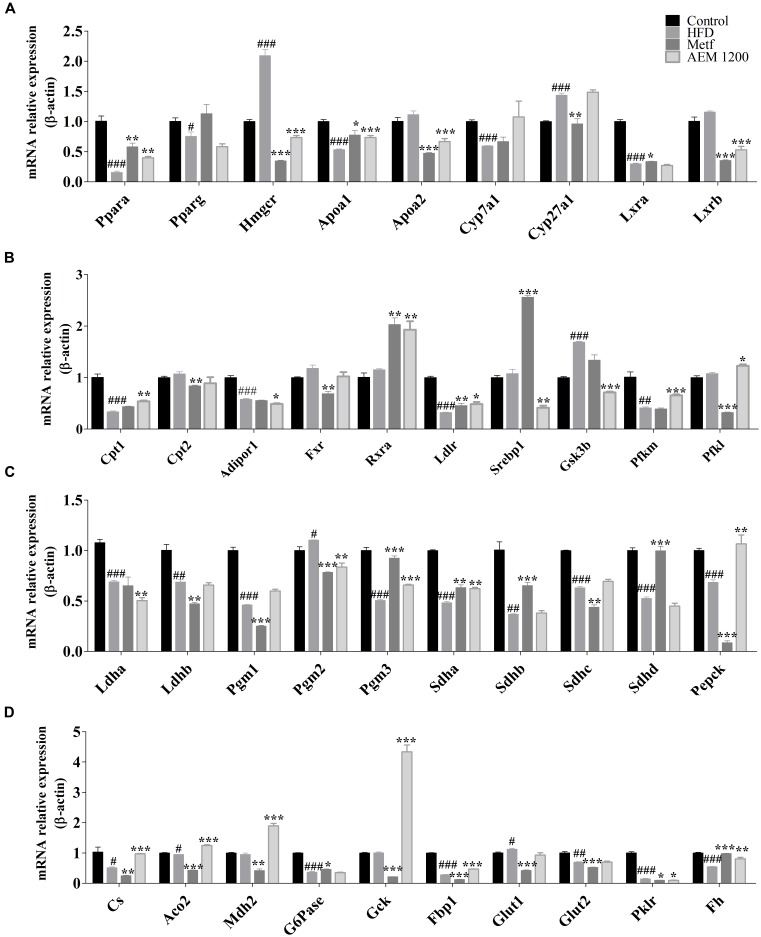
**(A–D)** mRNA expression levels of hepatic genes involved in lipid metabolism and the glycolysis/gluconeogenesis-TCA cycle. The mRNA levels were determined using quantitative real-time PCR and were normalized to β-actin mRNA expression. Data are presented as the means ± SEMs. ^#^*P* < 0.05, ^##^*P* < 0.01, ^###^*P* < 0.001 compared with the control group; ^∗^*P* < 0.05, ^∗∗^*P* < 0.01, ^∗∗∗^*P* < 0.001 compared with the HFD model group, *n* = 6.

To validate the aforementioned metabolic changes and *in silico* target prediction, the gene expression levels of key enzymes involved in glucose and lipid metabolism were assessed in liver tissues. PPARs, which serve as potential therapeutic targets for treating lipid metabolism disorder, and their downstream genes are involved in almost all aspects of lipid metabolism. As shown in **Figure 4**, the HFD feeding markedly reduced the hepatic mRNA levels of *Ppar*α and its target genes. In contrast, AEM (1,200 mg/kg) supplementation upregulated the hepatic mRNA levels of *Ppar*α (0.20-fold, *P* < 0.01), carnitine palmitoyltransferase 1 (*Cpt1*, 0.54-fold, *P* < 0.01), *Apoa1* (0.35-fold, *P* < 0.001), oxysterols receptor LXR-beta (*Lxrb*, 0.53-fold, *P* < 0.001), retinoic acid receptor alpha (*Rxra*, 1.92-fold, *P* < 0.01), and low-density lipoprotein receptor (*Ldlr*, 0.48-fold, P < 0.05), yet downregulated 3-hydroxy-3-methylglutaryl-coenzyme A reductase (*Hmgcr*, 0.73-fold, *P* < 0.001) and sterol regulatory element-binding protein 1 (*Srebp1*, 0.41-fold, *P* < 0.01). In addition, we checked the expression of selected genes involved in glycolysis/gluconeogenesis and the TCA cycle. AEM (1,200 mg/kg) upregulated the mRNA levels inhibited by the HFD, including ATP-dependent 6-phosphofructokinase (*Pfkl*, 1.23-fold, *P* < 0.05), phosphoglucomutases 2 and 3 (*Pgm2*, 0.84-fold, *P* < 0.01; *Pgm3*, 0.68-fold, *P* < 0.01), the succinate dehydrogenase complex, subunit A (*Sdha*, 0.73-fold, *P* < 0.01), phosphoenolpyruvate carboxykinase 1 (*Pepck*, 1.06-fold, *P* < 0.01), citrate synthase (*Cs*, 0.94-fold, *P* < 0.001), fetal hematoma (*Fh*, 0.67-fold, *P* < 0.01), glucokinase (*Gck*, 3.36-fold, *P* < 0.001), phosphofructokinase (*Pfkm*, 0.88-fold, *P* < 0.001), malate dehydrogenase (*Mdh2*, 1.89-fold, *P* < 0.001), fructose bisphosphatase 1 (*Fbp1*, 0.47-fold, *P* < 0.001), and aconitate hydratase, mitochondrial (*Aco2*, 1.24-fold, *P* < 0.001). Moreover, the expression of glycogen synthase kinase 3 beta (*Gsk3b*), a key regulatory enzyme in glucose metabolism, was significantly increased in the HFD group (1.69-fold, *P* < 0.001) but was decreased in the AEM (1,200 mg/kg) group (0.71-fold, *P* < 0.001).

### Energy Metabolism

**FIGURE 5:**
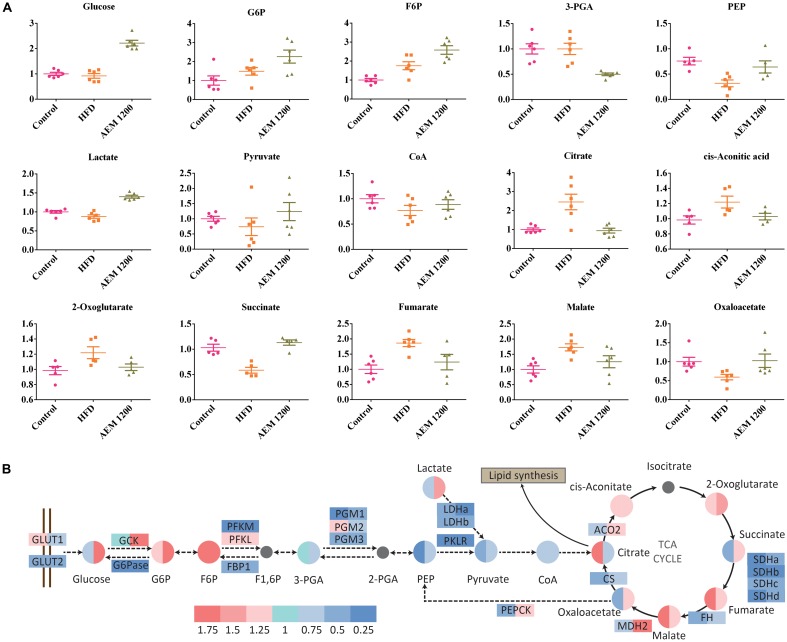
Aqueous extract of black maca (AEM) influences the metabolism of the glycolysis/gluconeogenesis pathway and the TCA cycle. **(A)** The scatter plot of the relative quantities of the energy metabolites in liver samples from the treated and untreated groups. The *Y*-axis data were normalized according to the average value of the control. **(B)** Differential analysis of the glycolysis/gluconeogenesis pathway and the tricarboxylic acid (TCA) cycle among the control group (Con), the HFD model group (HFD), and the 1,200 mg/kg AEM group (AEM 1200). Log_2_ fold changes of metabolites and the ratios of the average values of gene expression are color-coded, *n* = 5–6.

Metabolic disorder is closely associated with energy metabolism, including gluconeogenesis, glycogenolysis, and the TCA cycle. In the HFD group, the concentrations of the major TCA cycle intermediates increased. However, AEM (1,200 mg/kg) supplementation reversed the disorder induced by the HFD. Decreased concentrations of citrate (0.38-fold, *P* < 0.05), *cis*-aconitate (0.82-fold, *P* < 0.05), and fumarate (0.66-fold, *P* < 0.05) were observed in the liver tissue. Furthermore, the HFD also interfered with the glycolysis/gluconeogenesis pathway, as evidenced by the increased fructose 6-phosphate (F6P, 1.75-fold, *P* < 0.05) level but decreased phosphoenolpyruvic acid (PEP, 0.32-fold, *P* < 0.05) and lactate (0.88-fold, *P* < 0.05) levels. The F6P (1.47-fold, *P* < 0.05), lactate (1.60-fold, *P* < 0.001), oxaloacetate (1.73-fold, *P* < 0.05), and succinate (1.93-fold, *P* < 0.001) levels were higher in the AEM (1,200 mg/kg) treatment group than those in the HFD group (**Figure 5A**).

## Discussion

After 20 weeks, the golden hamster model of metabolism disorder induced by HFD feeding was established, with substantially increased serum levels of biochemical indicators (including TG, CHO, LDL-C, and others). The model was very appropriate because golden hamsters respond consistently to dietary modulation and, compared with the profiles of other rodents, showed a close similarity to human lipoprotein profiles (Liao et al., 2015). In this paper, we found that supplemental AEM (1,200 mg/kg) did not alter food intake but significantly prevented the increased in body weight, liver weight, and fat weight that occurred in golden hamsters fed a HFD for 20 weeks. This finding was consistent with previous reports in which maca was demonstrated to decrease body mass index (Gonzales, 2010). In addition, the food utilization rate was markedly lower in the AEM (600 and 1,200 mg/kg) groups than in the HFD group. These results indicated that AEM (1,200 mg/kg) may accelerate metabolism in golden hamsters.

The occurrence of lipid and glucose metabolism disorder is often accompanied by increased TG and CHO contents and is most directly associated with the LDL-C level. Vecera et al. (2007) found that maca had positive influences on lipid, antioxidative, and glucose parameters in hereditary hypertriglyceridemic rats. In this study, we also found that after 20 weeks, AEM administration (300, 600, and 1,200 mg/kg) dose-dependently decreased the serum CHO, TG, LDL-C, and insulin levels to near-baseline control values. Although the insulin level was significantly increased in the HFD group and was decreased in all the AEM treatment groups, the concentration of glucose did not change substantially in any group. This result may suggest that in the HFD group, the glucose level was regulated by a high insulin content, which is consistent with the lower insulin sensitivity index. In contrast, compared with the HFD-fed golden hamsters, the AEM-supplemented (600 and 1,200 mg/kg) hamsters presented with not only a lower insulin level but also improved insulin sensitivity. These findings indicated that AEM reduces HFD-induced hyperlipidemia and hyperinsulinemia and improves insulin sensitivity.

Liver is the central organ for lipid, carbohydrate, and protein metabolic processes, which are closely associated with metabolic diseases. Serum AST and ALT levels sensitively reflect hepatocyte damage, and TBIL represents the secretion and excretion function of the liver. In the HFD group, the hepatic coefficient and the hepatic levels of CHO, ALT, and TBIL increased markedly; in addition, the pathological section analysis revealed the presence of non-alcoholic fatty liver disease. Our results revealed that AEM (300, 600, and 1,200 mg/kg) treatment significantly reversed these trends and alleviated the lipid accumulation in the liver. The comprehensive results revealed that AEM improved liver secretion and excretion, protected liver cells, and reduced the levels of hepatic CHO and TG accumulation caused by the HFD.

Virtual screening is a computational technique used in drug discovery and is becoming increasingly popular in pharmaceutical research. The screening process searches libraries of small molecules to identify structures that are most likely to bind a drug target, typically a protein receptor or an enzyme (Rester, 2008). In this context, among the 16 identified targets, FBP1, ALDR, PPARγ, and GSK3B were the most promising maca targets for preventing and improving lipid and glucose metabolism disorder. In addition, the active compounds identified more targets, such as macaene (A14), which fished the protein targets FBP1, PTN1, PPARγ, and PPARα. These results are consistent with previous reports that macamides and macaenes are the main functional constituents of maca (Gonzales et al., 2005). With the *in silico* target fishing screening results, we identified targets related to glycolysis/gluconeogenesis pathways, including FBP1 and GSK3β (**Supplementary Table S2**). In addition, pathway analysis showed that AEM supplementation impacted the TCA cycle (**Figure 3A**). Considering the above results, we speculated that AEM prevented and improved metabolic impairments via the glycolysis/gluconeogenesis-TCA cycle (**Figure 5B**). Moreover, the expression profiles of genes involved in the glycolysis/gluconeogenesis-TCA cycle were measured (**Figure 4**).

The liver is a major player in maintaining glucose homeostasis, controlling the balance between glucose levels in the bloodstream and in hepatocytes (Jiang and Zhang, 2003). A key method for controlling hepatic glucose production and consumption is the net TCA-glycolytic/gluconeogenic flux. By regulating the activity levels of key enzymes, the liver can switch from net hepatic glucose consumption to glucose output. Although glycolysis and gluconeogenesis share several enzymes that catalyze reversible reactions, the irreversible key steps are catalyzed by separate enzymes subjected to different regulations. Therefore, regulating these gluconeogenic enzymes and their glycolytic counterparts by allosteric effectors, gene expression levels, or covalent modifications is a mechanism for achieving a net flux toward gluconeogenesis or glycolysis in the liver (Sharabi et al., 2015). Glucose is phosphorylated by *Gck*, a liver-specific hexokinase, to generate glucose 6-phosphate (G6P). *Gck* activity is regulated by its mRNA expression in hepatocytes (Peter et al., 2011). Compared with the HFD group, in the AEM (1,200 mg/kg) group, the mRNA expression level of *Gck* was increased more than twofold. The *Gck-*opposing gluconeogenic enzyme is *G6Pase*, and the HFD significantly decreased *G6Pase* expression. In addition, the highly significant accumulation of the G6P and F6P metabolic substrates in the AEM (1,200 mg/kg) group demonstrated that the dominant direction of the glucose/glucose-6-phosphate cycle was toward glycolytic flux. The F6P/F1, 6P substrate cycle is a major determinant of the net glycolytic or gluconeogenic flux. Thus, phosphofructokinase (*Pfkl* and *Pfkm*), the first committed step in glycolysis, together with its opposing gluconeogenic enzyme, *Fbp1*, controls the net glycolytic or gluconeogenic flux. Compared with the HFD group, the AEM (1,200 mg/kg) group presented with decreased *Fbp1* mRNA expression, while *Pfkm* gene expression was upregulated. The first step in glycogen breakdown is catalyzed by glycogen phosphorylase, and the final step in glycogen synthesis is catalyzed by glycogen synthase. GSK3 inhibits glycogen synthesis by suppressing glycogen synthase through inhibitory phosphorylation. Type 2 diabetes is strongly associated with decreases in insulin-stimulated glycogen synthase activity and glycogen synthesis and with increased GSK3 protein levels (Lee and Kim, 2007). Similarly, AEM (1,200 mg/kg) reversed the increased *Gsk3b* mRNA expression in the HFD group. In addition, lactate levels were increased, and the mRNA expression of *Ldha* was reduced in the AEM (1,200 mg/kg) group, which suggests an increased rate of glycolysis, consistent with a previous study (Chao et al., 2014; Le et al., 2016). Overall, these results indicated that AEM promotes glycolysis and inhibits gluconeogenesis.

The final products of fatty acid degradation and glycolysis are used in the TCA cycle (Friedrich, 2012). Due to the importance of this cycle in the central mitochondrial metabolic pathway, certain metabolic disturbances through this metabolic pathway have been reflected in obese and type 2 diabetic subjects (Satapati et al., 2012; Lu et al., 2013). Our metabolomic data revealed that the concentration change of six TCA cycle intermediates, citrate, 2-oxoglutarate, fumarate, malate, oxaloacetate, and succinate, were highly variable between the HFD and AEM (1,200 mg/kg) groups. Recently, Patterson et al. (2011) found that, compared with normal animals, type 2 diabetic rhesus macaques demonstrated a twofold higher level of citrate, which is consistent with our results. Furthermore, our qPCR results revealed that *Mdh2*, *Fh*, *Aco2*, *Sdha*, *Sdhb*, *Sdhd*, and *Cs* levels were downregulated in the HFD-fed golden hamsters, which likely contributed to the substantial malate, fumarate, and citrate accumulation. However, compared with the HFD group, the AEM (1,200 mg/kg)-treated group demonstrated lower levels of citrate, malate, and fumarate and high mRNA expression levels of *Sdha*, *Sdhd*, *Fh*, *Mdh2*, *Aco2*, and *Cs*. This result suggested that AEM increased TCA cycle flux by upregulating *Aco2*, *Fh*, *Cs*, *Sdha*, and *Mdh2* gene expression. Some studies have indicated that a reduced TCA cycle flux may be a metabolic adaptation that occurs during the metabolic progression of normal rats to diabetic phenotypes (Bakar et al., 2015; Zhou et al., 2015). In summary, via an array of mechanisms, our results indicated that AEM improves glucose metabolism disorder, specifically by upregulating the TCA cycle pathway.

From the biochemical data, we found that AEM affected hyperlipidemia, and the *in silico* target prediction identified genes related to lipid metabolism, especially PPARs. To elucidate the mechanism underlying the hypolipidemic effect of AEM, we assessed the expression profiles of hepatic genes involved in lipid metabolism. An increasing amount of evidence suggests that *Ppar*α is a key regulator of lipid metabolism. Known *Ppar*α target genes are involved in almost all aspects of lipid metabolism, including fatty acid uptake, binding, and oxidation; lipoprotein assembly; and lipid transport (Neve et al., 2000). Increases in the mRNA expression levels of *Ppar*α and its target genes have also been identified as a mechanism for improving glucose and lipid metabolic disorders in high-fat diet-fed mice (Ding et al., 2013). In the AEM (1,200 mg/kg) group, the mRNA expression levels of *Ppar*α and its downstream genes, including *Rxr* and *Cpt1*, were substantially upregulated, but *Hmgcr* and *Lxrb* were downregulated. Increased fatty acid β-oxidation leads to decreased serum TG concentrations in rodents (Reddy and Rao, 2005). In the AEM (1,200 mg/kg) consumption group, the increased *Ppar*α mRNA expression upregulated the expression of *Cpt1* mRNA, which modulates fatty acid β-oxidation. This finding showed that AEM-reduced lipid synthesis may contribute to fatty acid β-oxidation. Positive correlations between *Lxrb* expression and the degrees of lipid accumulation, inflammation, and fibrosis in the livers of patients with non-alcoholic fatty liver have also been noted (Ahn et al., 2014). Thus, inhibiting the expression of *Lxrb* and its target genes reversed the high serum TG levels and improved steatosis in HFD-fed mice (Ding et al., 2012). *Srebp1c* plays a critical role in synthesizing lipids, suggesting that SREBP pathway inhibition might be a potential approach for treating obesity (Xiao and Song, 2013). In this paper, AEM reduced the expression levels of *Lxrb* and its target gene, *Srebp1*. In addition, the most important change at the mRNA level was found in the expression of *Hmgcr*, a rate-limiting enzyme in CHO synthesis. AEM (1,200 mg/kg) significantly reversed the increased *Hmgcr* transcript level in HFD-fed golden hamsters and, therefore, may reduce CHO synthesis. In summary, the above results are consistent with the lower serum and liver levels of CHO and TGs and demonstrate that the lipid-lowering effect of AEM may be partly related to the enhanced expression of genes involved in lipid metabolism through *Ppar*α activation, fatty acid β-oxidation, and lipogenesis inhibition.

Limitations exist in this work. First, the studies on chemical compositions and the quantitative analysis were not sufficiently deep, restricting the comprehensiveness of the database used by virtual screening. In addition, further work is needed to determine the prevention effectiveness of AEM on metabolism disorder in clinical trials.

## Conclusion

Our study first demonstrated that AEM supplementation positively affected lipid and glucose metabolism disorder prevention and amelioration in hamsters. A golden hamster model of metabolism disorder induced by feeding HFD was established, which shared many similarities with human lipid metabolism. Based on the serum biochemical and histopathological results, AEM appeared to significantly prevent the hyperlipidemia, hyperinsulinemia, insulin resistance, and non-alcoholic fatty liver caused by the HFD. For the first time, metabolomics, virtual screening, and qPCR were used together to reveal the complex pathogenesis underlying the protective effect of AEM on metabolic impairments. AEM promoted glycolysis and inhibited gluconeogenesis via regulating the expression levels of key genes such as *Gck*, *Fbp1*, and *Pfkm.* Moreover, AEM upregulated TCA cycle flux activity by changing the intermediate concentrations and increasing the mRNA levels of *Aco2*, *Fh*, *Cs*, *Sdha*, and *Mdh2.* In addition, the lipid-lowering effects of AEM in both the serum and liver may be partly related to PPARα signaling activation, including enhanced fatty acid β-oxidation and lipogenesis pathway inhibition. Together, our data demonstrated that the AEM intervention significantly improved metabolism disorder by regulating the glycolysis/gluconeogenesis-TCA cycle pathway and via modulating the expression levels of genes involved in the PPARα signaling pathway. Thus, AEM has the potential to be a dietary supplement for preventing or at least slowing lipid and glucose metabolism disorder progression. However, more studies and clinical trials are needed to support this hypothesis.

## Author Contributions

WW, LX, BJ, and PX: designed the experiments, supervised, and participated in the entire work. WW, HL, and JX: maintained and performed animal studies, biochemical analysis, metabonomics, and qPCR analysis. FY, JX, and HL: supported *in silico* analysis. WW, FY, and BJ: contributed to data analysis and writing and editing the manuscript. All the authors read and approved the final manuscript.

## Conflict of Interest Statement

The authors declare that the research was conducted in the absence of any commercial or financial relationships that could be construed as a potential conflict of interest.

## Footnotes

1 http://www.metaboanalyst.ca

2 http://www.daylight.com/smiles

## Supplementary Material

FIGURE S1 Effects of aqueous extract of black maca (AEM) on the food intake and body weight of golden hamsters over 20 weeks. **(A)** Daily food intake of each golden hamster. **(B)** The body weight of each golden hamster over the 20 weeks. Data are presented as the means ± SEMs, *n* = 6.

FIGURE S2 Number of epididymal white adipocytes per field of vision. The number of epididymal white adipocytes per field of vision was measured. Three fields of vision of each pathology sections were selected randomly. Each field of vision was observed at a magnification of 400×, *n* = 4.

FIGURE S3 Effects of aqueous extract of black maca (AEM) on serum lipid profiles and glucose and insulin levels in golden hamsters over 20 weeks. **(A)** At the end of 4 weeks, serum CHO, TG, LDL, HDL, and glucose levels were tested. **(B)** At the end of 8 weeks, serum CHO, TG, LDL, HDL, and glucose levels were measured. **(C)** At the end of 12 weeks, serum CHO, TG, LDL, HDL, and glucose levels were tested. **(D)** At the end of 16 weeks, serum CHO, TG, LDL, HDL, and glucose levels were measured. Data are presented as the means ± SEMs. ^#^*P* < 0.05, ^##^*P* < 0.01, ^###^*P* < 0.001 compared with the control group; ^∗^*P* < 0.05, ^∗∗^*P* < 0.01, ^∗∗∗^*P* < 0.001 compared with the HFD model group, *n* = 6.

TABLE S1 Hamster quantitative real-time PCR primer sequences.

TABLE S2 Major compound-target-disease table for maca.

FILE S1 The collection of chemical constituents of maca.
