# Supplementary figures and images for: Aqueous Extract of Black Maca Prevents Metabolism Disorder via Regulating the Glycolysis/Gluconeogenesis-TCA Cycle and PPARα Signaling Activation in Golden Hamsters Fed a High-Fat, High-Fructose Diet
Source: Front Pharmacol. 2018 Apr 6;9:333. doi: 10.3389/fphar.2018.00333 (PMC5897445; doi:10.3389/fphar.2018.00333)

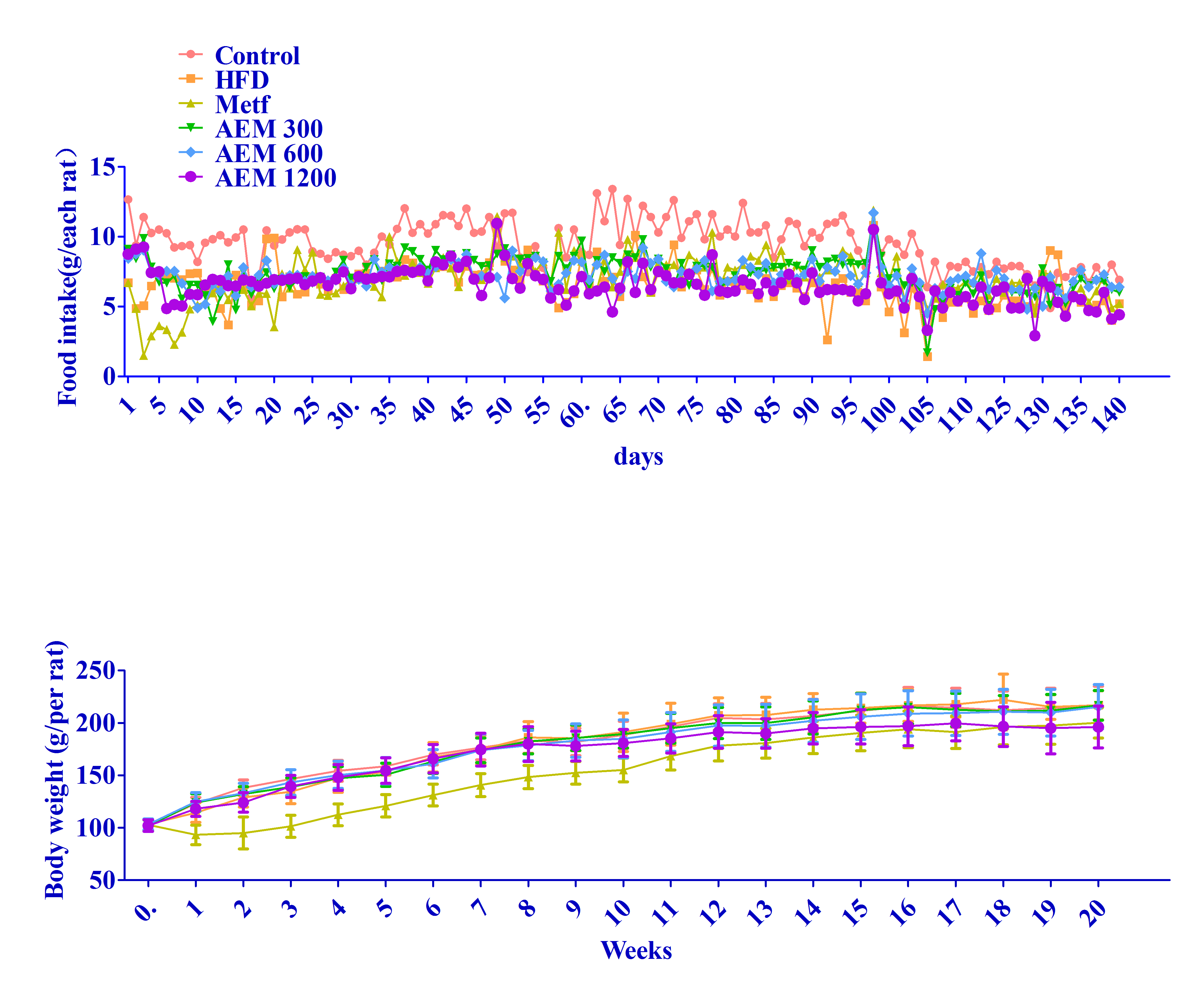

Supplement: FIGURE S1 — Effects of aqueous extract of black maca (AEM) on the food intake and body weight of golden hamsters over 20 weeks. (A) Daily food intake of each golden hamster. (B) The body weight of each golden hamster over the 20 weeks. Data are presented as the means ± SEMs, n = 6. [file Image_1.TIF]

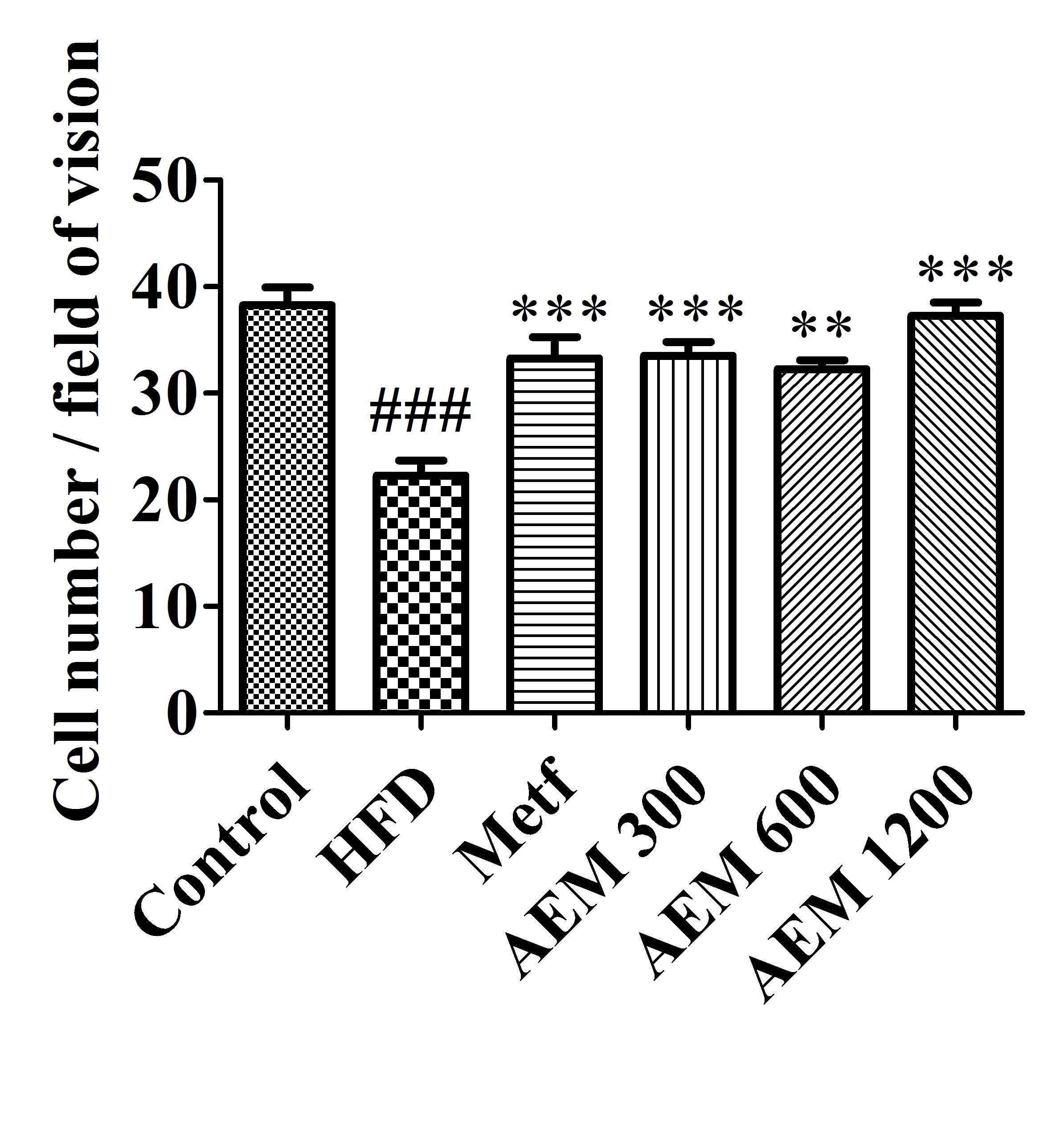

Supplement: FIGURE S2 — Number of epididymal white adipocytes per field of vision. The number of epididymal white adipocytes per field of vision was measured. Three fields of vision of each pathology sections were selected randomly. Each field of vision was observed at a magnification of 400×, n = 4. [file Image_2.TIF]

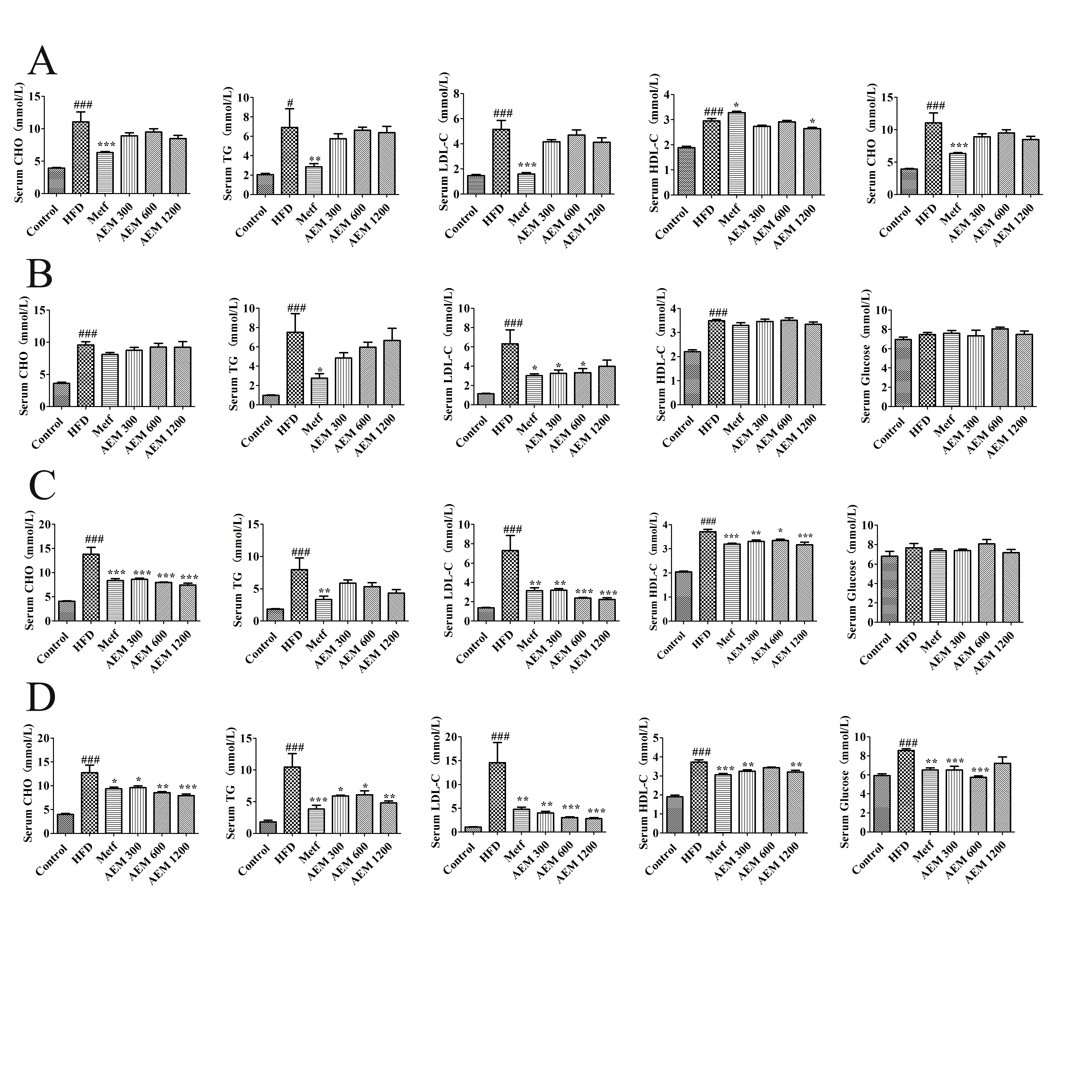

Supplement: FIGURE S3 — Effects of aqueous extract of black maca (AEM) on serum lipid profiles and glucose and insulin levels in golden hamsters over 20 weeks. (A) At the end of 4 weeks, serum CHO, TG, LDL, HDL, and glucose levels were tested. (B) At the end of 8 weeks, serum CHO, TG, LDL, HDL, and glucose levels were measured. (C) At the end of 12 weeks, serum CHO, TG, LDL, HDL, and glucose levels were tested. (D) At the end of 16 weeks, serum CHO, TG, LDL, HDL, and glucose levels were measured. Data are presented as the means ± SEMs. #P < 0.05, ##P < 0.01, ###P < 0.001 compared with the control group; ∗P < 0.05, ∗∗P < 0.01, ∗∗∗P < 0.001 compared with the HFD model group, n = 6. [file Image_3.TIF]
