# Supplementary material for: Aqueous Extract of Black Maca Prevents Metabolism Disorder via Regulating the Glycolysis/Gluconeogenesis-TCA Cycle and PPARα Signaling Activation in Golden Hamsters Fed a High-Fat, High-Fructose Diet
Source: Front Pharmacol. 2018 Apr 6;9:333. doi: 10.3389/fphar.2018.00333 (PMC5897445; doi:10.3389/fphar.2018.00333)
Supplement: FILE S1 — The collection of chemical constituents of maca. [file Data_Sheet_1.DOCX]

**File S1. The collection of chemical constituents of maca.**

| No. | Structure |
| --- | --- |
| A-1 |  |
| A-2 |  |
| A-3 |  |
| A-4 |  |
| A-5 |  |
| A-6 |  |
| A-7 |  |
| A-8 |  |
| A-9 |  |
| A-10 |  |
| A-11 |  |
| A-12 |  |
| A-13 |  |
| A-14 |  |
| A-15 |  |
| A-16 |  |
| A-17 |  |
| A-18 |  |
| A-19 |  |
| A-20 |  |
| A-21 |  |
| A-22 |  |
| A-23 |  |
| A-24 |  |
| A-25 |  |
| A-26 |  |
| A-27 |  |
| A-28 |  |
| A-29 |  |
| A-30 |  |
| A-31 |  |
| A-32 |  |
| A-33 |  |
| A-34 | **** |
| A-35 | **** |
| J1 |  |
| J2 |  |
| J3 |  |
| J4 |  |
| J5 |  |
| J6 |  |
| J7 |  |
| J8 |  |
| J9 |  |
| J10 |  |
| J11 |  |
| J12 |  |
| J13 |  |
| J14 |  |
| J15 |  |
| J16 |  |
| J17 |  |
| J18 |  |
| J19 |  |
| J20 |  |
| J21 |  |
| S1 |  |
| S2 |  |
| S3 |  |
| S4 |  |
| S5 |  |
| S6 |  |
| S7 |  |
